# Resource competition-driven bistability and stochastic switching amplify gene expression noise

**DOI:** 10.1371/journal.pcbi.1012931

**Published:** 2025-04-23

**Authors:** Hanah Goetz, Rong Zhang, Xiao Wang, Xiao-Jun Tian

**Affiliations:** School of Biological and Health Systems Engineering, Arizona State University, Tempe, Arizona, United States of America; Max Planck Institute for Evolutionary Biology, GERMANY

## Abstract

Although the impact of resource competition on the deterministic behavior of synthetic gene circuits has been studied, its effects on gene expression noise remain obscure. In this work, we systematically analyze the role of resource competition in noise propagation within a genetic inhibition cascade circuit. We found that resource competition amplifies gene expression noise by introducing unexpected bistability and stochastic switching between the two stable states. This emergent bistability, driven by resource competition-mediated double negative feedback, allows one gene to dominate expression while suppressing the other in a “winner-takes-all” behavior. Our findings highlight the critical role of resource competition in shaping the noise dynamics and its propagation, underscoring the importance of considering these effects when designing and controlling synthetic circuits.

## Summary

In this work, we investigate how competition for shared cellular resources affects the reliability and uncertainty of synthetic gene circuits, which are engineered programs within cells. While recent research focuses more on “deterministic” effects on gene circuits under limited resources, their impact on the fluctuations or “noise” in gene activity is less explored. We found that when two genes in a cascade compete for shared resources, unexpected feedback loops can emerge, allowing one gene to temporarily dominate and suppress the other in a winner-takes-all effect. This competition can create bistability, where the system switches stochastically between two stable states due to resource-sharing constraints. Our findings reveal that resource competition introduces an additional layer of unpredictability to gene circuits, with implications for fields like gene therapy and biomanufacturing. By accounting for these effects, we can design engineered cells to be more reliable and consistent for future applications.

## Introduction

It is now well recognized that cellular resources, such as RNA polymerase for transcription and ribosomes for translation, are highly limited for synthetic gene circuits. Consequently, competition for these resources often leads to unintended coupling between genes and modules, making the engineering process challenging and system behavior unpredictable [1–12]. Studies have shown that the expression levels of two independent genes are negatively correlated in a linear fashion, following isocost lines or analogously, Ohm’s law, due to resource competition [13,14]. In genetic activation cascade systems, the expected monotonic dose-response curve becomes nonmonotonic as resource competition causes the upstream gene to inhibit the downstream gene [15]. Interestingly, “winner-takes-all” (WTA) behavior has been observed in systems with two independent or mutually positively regulated self-activating modules [16]. While the impact of resource competition on the deterministic behavior of gene circuits has been explored, its effect on the noise behavior of these circuits remains not fully understood.

Since resource competition directly impacts transcription and translation, it is essential to consider it when evaluating system noise. Resource competition adds a new layer of complexity to analyzing gene expression noise. Previous studies have highlighted the challenges in measuring noise and understanding it roles in shaping gene circuit behavior [17–19]. Recently, we discovered that resource competition plays a double-edged role in the gene expression noise of a two-gene system [20]. While resource limitation can dampen fluctuations in gene expression, reducing noise, resource competition also introduces a new source of noise, as expression fluctuations in one gene affect resource availability for the other gene. However, the impact of resource competition on stochastic effects in more complex synthetic gene circuits remains largely unexplored, representing a significant gap in our understanding of noise dynamics in these systems.

Here, we focus on noise propagation in a genetic inhibition cascade system. Previous studies have shown that longer transcriptional cascades increase steady-state sensitivity and amplify cell-to-cell variability in intermediate regions of expression [21]. Noise in a gene can arise from intrinsic fluctuations, transmitted noise from upstream genes, and global noise affecting all genes in the system [22]. In this work, we incorporate resource competition into our analysis of noise propagation in a genetic inhibition cascade. By examining system dynamics under unlimited, limited, and orthogonal resource conditions, we explore how resource competition impacts the stability and noise characteristics of gene circuits.

## Results

### Noise propagation under the assumption of unlimited resources

**Fig 1:**
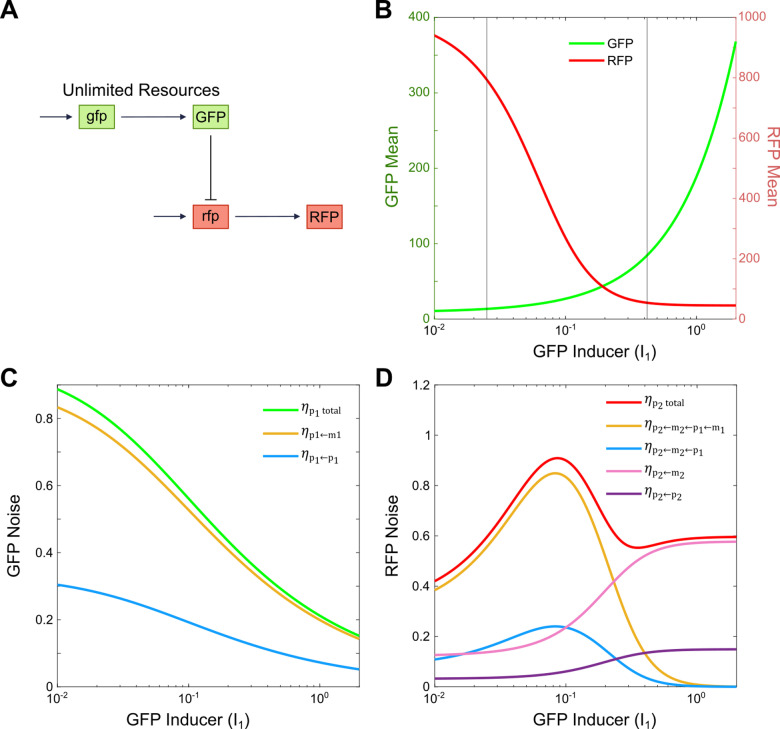
Noise propagation in a genetic inhibition cascade circuit with unlimited resources. A) Schematic of a genetic inhibition cascade circuit where GFP, expressed from the gfp gene, inhibits the transcription of RFP under conditions of unlimited transcriptional and translational resources. (B) Mean expression levels of GFP and RFP as a function of increasing GFP inducer dose ($I_{1}$). C) Total noise level in GFP expression and its two noise components plotted against GFP inducer dose ($I_{1}$). D) Total noise level in RFP expression and its four noise components as a function of GFP inducer dose ($I_{1}$).

To investigate noise propagation, we developed multiple models for a two-gene cascade circuit (see Method for details). In this system, one gene (GFP) inhibits another (RFP), as illustrated in **Fig 1A**. We used GFP and RFP to represent two genes for simplicity in this theoretical work. In an experimental setup to validate the prediction from this work, the system would also require transcription factors to implement transcriptional inhibition. In all models, we utilized both deterministic and stochastic equations to simulate the dynamics of mRNA and protein concentrations or counts for two genes. The production rates of the two mRNAs were modeled using Hill functions, which depend on the inducer dose (I_i_). We have considered three different conditions: unlimited resources, limited resource and orthogonal resource. Resource competition was modeled using a partition function for the transcription and translation rates, as described in our previous works.

We began by analyzing the model under the assumption of unlimited resources, a common baseline in previous noise propagation studies. The dose-response curve in **Figs 1B** and S1 shows that increasing the GFP inducer dose leads to a rise in GFP mRNA and protein mean expression, alongside a sigmoidal decrease in RFP mRNA and protein mean expression, reflecting the nonlinear inhibition exerted by GFP on RFP. To further understand the system’s noise behavior, we derived the analytical solution for the noise levels of the two proteins (see Method Section for details). The total noise in GFP is given by the following equation:


$$\eta_{p_{1}\;total}^{2}=\overset{\eta_{p_{1}\leftarrow p_{1}}^{2}}{\overbrace{\frac{1}{P_{1}}}}+\overset{\eta_{p_{1}\leftarrow m_{1}}^{2}}{\overbrace{\eta_{m_{1}\leftarrow m_{1}}^{2}H_{21}^{2}\frac{\frac{1}{\tau_{2}}}{\frac{1}{\tau_{1}}+\frac{1}{\tau_{2}}}}},$$

Where ${\;\eta }_{m_{1}\leftarrow m_{1}}^{2}=\frac{1}{M_{1}}$.

Here, P1 and M1 are the mean numbers of GFP protein and mRNA, respectively. The first term, $\eta_{p_{1}\leftarrow p_{1}}^{2}$, depends on the mean number of GFP protein, capturing the noise due to the fluctuation from GFP birth and death. The second term, $\eta_{p_{2}\leftarrow m_{1}}^{2}$, depends on the mean number of GFP mRNA, representing the noise propagated from GFP mRNA, resulting from its birth and death. The variables $H_{ij}$ represent static susceptibility, and terms including $\tau_{i}$ are meant to show time-averaging, where indices i and j correspond to the positions in the cascade for either mRNA or protein.

As the GFP inducer dose increases, total GFP noise decreases (**Fig 1C**), which aligns with the increase in GFP mean expression (**Fig 1B**). The noise propagated from GFP mRNA has the greatest effect on the GFP noise level, rather than noise originating from GFP protein birth and death, as demonstrated by the comparison between the yellow and blue curves in **Fig 1C**. This is largely due to the smaller number of GFP mRNA molecules, making translation a noisier process compared to the birth and death dynamics of GFP protein.

Interestingly, the total noise in RFP is given by the following equation:


$$\eta_{p_{2}\;total}^{2}=\overset{\eta_{p_{2}\leftarrow p_{2}}^{2}}{\overbrace{\frac{1}{P_{2}}}}+\overset{\eta_{p_{2}\leftarrow m_{2}}^{2}}{\overbrace{\eta_{m_{2}\leftarrow m_{2}}^{2}H_{43}^{2}\frac{\frac{1}{\tau_{4}}}{\frac{1}{\tau_{3}}+\frac{1}{\tau_{4}}}}}+\overset{\eta_{p_{2}\leftarrow m_{2}\leftarrow p_{1}}^{2}}{\overbrace{\eta_{m_{2}\leftarrow p_{1}}^{2}\left(1+\frac{\frac{1}{\tau_{3}}}{\frac{1}{\tau_{2}}+\frac{1}{\tau_{4}}}\right)H_{43}^{2}\frac{\frac{1}{\tau_{4}}}{\frac{1}{\tau_{3}}+\frac{1}{\tau_{4}}}}}+$$

$$\overset{\eta_{p_{2}\leftarrow m_{2}\leftarrow p_{1}\leftarrow m_{1}}^{2}}{\overbrace{\eta_{m_{2}\leftarrow p_{1}\leftarrow m_{1}}^{2}\left(1+\frac{\frac{1}{\tau_{3}}}{\frac{1}{\tau_{2}}+\frac{1}{\tau_{4}}}\left(1+\frac{\frac{1}{\tau_{2}}}{\frac{1}{\tau_{1}}+\frac{1}{\tau_{2}}+\frac{1}{\tau_{3}}}\cdot \frac{\frac{1}{\tau_{2}}+\frac{1}{\tau_{3}}}{\frac{1}{\tau_{1}}+\frac{1}{\tau_{4}}}\right)\right)H_{43}^{2}\frac{\frac{1}{\tau_{4}}}{\frac{1}{\tau_{3}}+\frac{1}{\tau_{4}}}}}$$

where


$$\eta_{m_{2}\leftarrow m_{2}}^{2}=\frac{1}{M_{2}}$$

$$\eta_{m_{2}\leftarrow p_{1}}^{2}=\eta_{p_{1}\leftarrow p_{1}}^{2}H_{32}^{2}\frac{\frac{1}{\tau_{3}}}{\frac{1}{\tau_{2}}+\frac{1}{\tau_{3}}}$$

$$\eta_{m_{2}\leftarrow p_{1}\leftarrow m_{1}}^{2}=\eta_{p_{1}\leftarrow m_{1}}^{2}\left(1+\frac{\frac{1}{\tau_{2}}}{\frac{1}{\tau_{1}}+\frac{1}{\tau_{3}}}\right)H_{32}^{2}\frac{\frac{1}{\tau_{3}}}{\frac{1}{\tau_{2}}+\frac{1}{\tau_{3}}}$$

Here, P2 and M2 are the mean numbers of RFP protein and mRNA, respectively. The first term, $\eta_{p_{2}\leftarrow p_{2}}^{2}$, depends on the mean number of RFP protein, capturing the noise from the fluctuation due to stochastic RFP protein birth and death. The second term, $\eta_{p_{2}\leftarrow m_{2}}^{2}$, depends on the mean number of RFP mRNA, representing noise propagated from RFP mRNA due to its birth and death. The third term, $\eta_{p_{2}\leftarrow m_{2}\leftarrow p_{1}}^{2}$, depends on the mean number of GFP protein, representing the noise propagated from the GFP protein birth and death. Lastly, the fourth term, $\eta_{p_{2}\leftarrow m_{2}\leftarrow p_{1}\leftarrow m_{1}}^{2}$, depends on the mean number of GFP mRNA and captures the noise propagated from GFP mRNA birth and death noise, transmitted through both GFP protein and RFP mRNA in the cascade.

**Fig 1D** shows the dependence of RFP total noise on increasing GFP inducer dose. Interestingly, the RFP noise curve exhibits a nonmonotonic behavior, with a hump appearing at intermediate GFP inducer dose. This is consistent with previous experiment data [22]. To understand this, we analyzed the contributions of each noise component to RFP noise (also shown in **Fig 1D**). Near the inhibition threshold, where GFP strongly affects RFP, noise propagated from GFP mRNA (yellow line, $\eta_{p_{2}\leftarrow m_{2}\leftarrow p_{1}\leftarrow m_{1}}^{2}$) and GFP protein (blue line, $\eta_{p_{2}\leftarrow m_{2}\leftarrow p_{1}}^{2}$) have a major impact on RFP total noise, creating the observed hump. This results from the high sensitivity of RFP to GFP fluctuations around the inhibition threshold. Around this point, a slight increase in GFP inducer dose leads to a small increase in GFP mRNA and protein levels, which however triggers a large decrease in RFP mRNA and protein expression (**Figs 1B** and S1). Consequently, noise components originating from GFP mRNA and protein have a pronounced effect on RFP noise at low GFP inducer dose. However, as GFP inducer dose increases and RFP becomes nearly fully inhibited by GFP, these contributions from GFP-derived noise decrease. At high GFP inducer dose, the RFP-associated terms, $\eta_{p_{2}\leftarrow m_{2}}^{2}$ and $\eta_{p_{2}\leftarrow p_{2}}^{2}$begin to dominate RFP noise due to the low copy numbers of RFP mRNA and protein. Thus, post-inhibition, these RFP-specific noise terms become most significant. Stronger or weaker dissociation constant of GFP-mediated inhibition on RFP translation shifts this pattern without altering the qualitative behavior, as shown in S2 Fig.

### Noise propagation and stochastic state switching under resource competition

**Fig 2:**
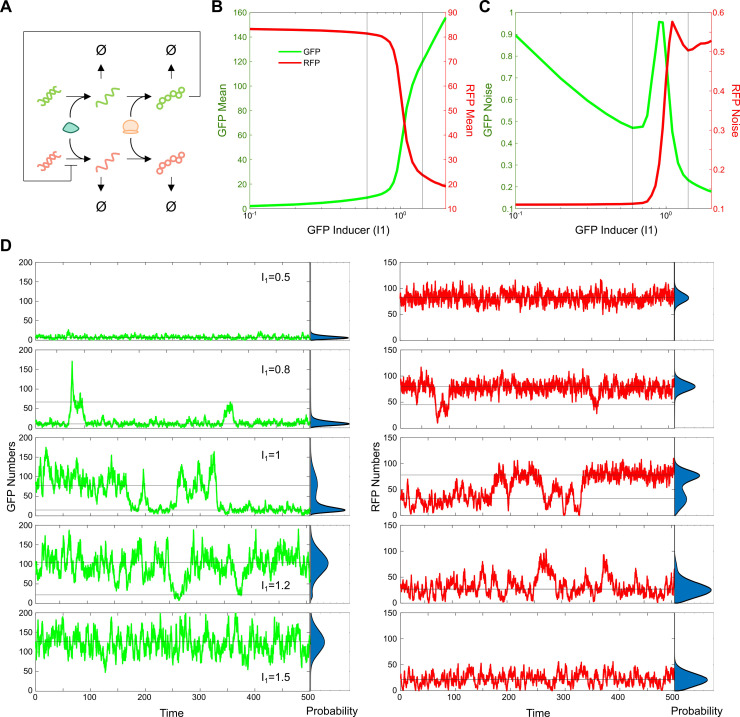
Resource competition induces stochastic state switching, impacting noise propagation. A) Schematic of genetic inhibition cascade circuit when the two genes share transcriptional resources (RNAPs) for mRNA synthesis and translational resources (ribosomes) for protein synthesis. B) Mean protein expression levels for GFP and RFP from stochastic simulation as a function of increasing GFP inducer dose ($I_{1}$). C) Total noise levels for GFP and RFP as GFP inducer dose ($I_{1}$) increases. Vertical lines represent inflection points for GFP and RFP mean curves, acting as inhibition threshold guidelines. D) Stochastic trajectories showing GFP and RFP protein levels across different GFP inducer doses ($I_{1}$). with horizontal lines indicating the local protein mode(s). Probability distributions of protein counts are shown alongside each trajectory plot.

We further analyzed the cascade system under limited resources, where RNA polymerases (RNAPs) and ribosomes are shared between genes for transcription and translation, respectively, as illustrated in **Fig 2A**. The pattern of mean protein expression as GFP inducer dose ($I_{1}$) increases is similar to the case with unlimited resources (**Fig 2B**). While the RFP curve still exhibits a reverse sigmoidal shape, the inhibition threshold is significantly higher compared to the unlimited resource case. This is expected in the context of resource com, as GFP must first compete with RFP for limited resources before it can inhibit RFP. As a result of this additional resistance from resource competition, a higher GFP inducer dose is required to effectively inhibit RFP. Moreover, the GFP curve also takes on a sigmoidal shape, with a very slow increase at low $I_{1}$ levels due to resource competition with RFP, and saturation at high $I_{1}$ levels due to resource limitation.

In this scenario, it is not feasible to obtain an analytical solution for the protein noise levels as here resource competition adds significant complexity to the system. Instead, we employed Gillespie stochastic simulations (see Method Section for details) to investigate the system’s noise behavior. As shown in **Fig 2C**, Interestingly, GFP noise exhibits a non-monotonic pattern as GFP inducer dose increases. Initially, it decreases similarly to the system without resource competition, but then rises rapidly to a peak near $I_{1}=0.87$ before decreasing again. RFP noise remains relatively stable up to $I_{1}=0.87$, but then increases sharply, reaching a peak at $I_{1}=1.1$ before decreasing and then leveling off. This implies that protein expression near these thresholds experiences high fluctuations around the mean.

To understand the underlying cause, we investigated the stochastic trajectories at different GFP inducer doses (**Fig 2D**). At a low GFP inducer dose ($I_{1}=0.5$, top panels), the system behaves as expected; GFP expression is low, fluctuating around a small mode (the local maximums in the probability distribution), while RFP expression is high with fluctuations around a high mode. Both GFP and RFP exhibit unimodal distributions. With a slight increase in GFP inducer dose ($I_{1}=0.8$), fluctuations in both GFP and RFP increase around their respective low and high modes, creating additional local high and low modes. However, once the first threshold is surpassed ($I_{1}=1$), both GFP and RFP show bimodal distribution with two distinct peaks, signaling the presence of two stable states in the system. GFP and RFP fluctuate between these two states. Interestingly, when GFP fluctuates around the high state, RFP is around the low state, or vice versa, showing a WTA behavior as observed in previous work [16]. After passing the second GFP inducer dose threshold ($I_{1}=1.5$), the system returns to unimodal distribution, with GFP fluctuating around a high mode and RFP fluctuating around a low mode instead. These results suggest that there is a region of high noise in the system, driven by the emergence of a bimodal distribution and stochastic switching between the two stable states due to resource competition.

We observed similar behavior when varying the dissociation constant of GFP-mediated inhibition on RFP translation, as shown in S3 Fig Both the dose-response and noise curves as functions of the dissociation constant follow a comparable pattern. Here, an increase in the inhibition dissociation constant makes the inhibition of RFP by GFP less effective. With a smaller inhibition threshold, GFP expression is high, and RFP expression is low, indicating that direct inhibition of RFP by GFP outweighs any indirect effects from resource competition. However, with a larger inhibition dissociation constant, RFP expression is high and GFP is low, suggesting that resource competition now favors RFP, mitigating GFP’s inhibitory effect. Similarly, the enhanced noise level was observed, as the fluctuations between high and low modes in the two proteins create amplified variability in the system.

### Resource competition-induced bistability drives stochastic state switching

**Fig 3:**
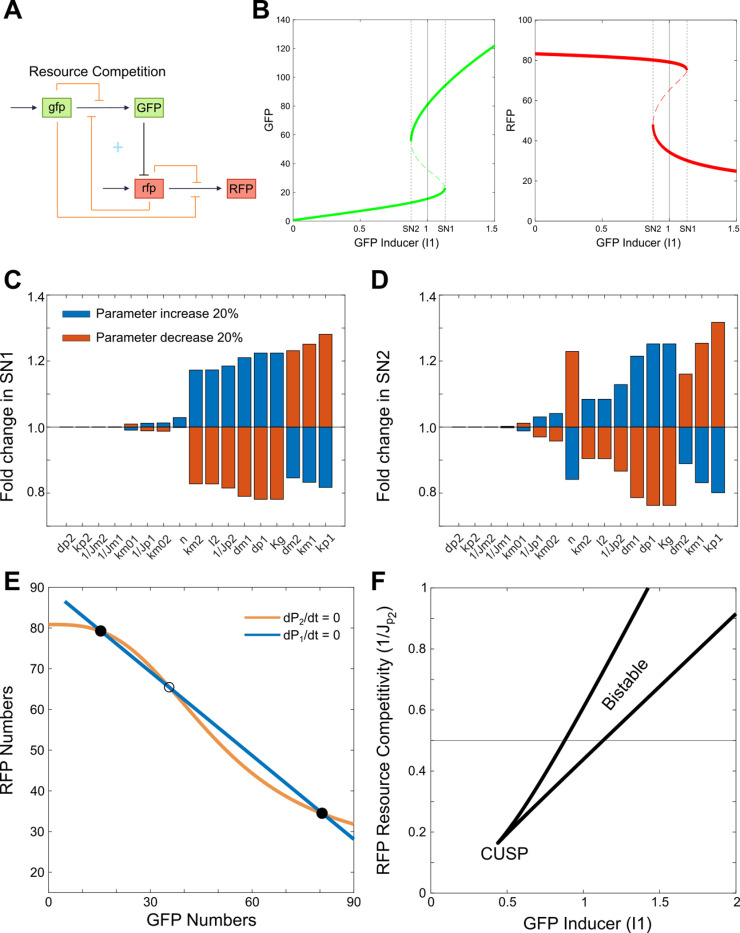
Resource competition introduces an implicit positive feedback loop, enabling bistability in the system. A) Schematic of genetic inhibition cascade circuit with hidden links (orange links) mediated by resource competition. A double negative feedback loop (indicated by the blue ‘+’) is shown between GFP and RFP. B) One-parameter bifurcation analysis showing bistability of GFP (left) and RFP (right) protein levels as the GFP inducer dose ($I_{1}$) varies. The vertical solid line represents the I_1_ value used in panel E, while the vertical dashed lines indicate the two saddle-node bifurcation points. C-D) The percent changes in I_1_ values at the saddle-node bifurcation points (SN1 and SN2) when each parameter is increased or decreased by 20% relative to the standard parameter set. E) Nullcline analysis of GFP and RFP shows intersections that create two stable steady states (solid circles) and one unstable steady state (open circle). Each nullcline represents the interdependence of two variables when the rate of change for one variable in the system is zero. F) Two-parameter bifurcation diagram indicating the saddle-node bifurcation points as GFP inducer dose ($I_{1}$) and RFP resource competitivity ($1/J_{p_{2}}$) vary. The two lines here represent the saddle-node bifurcation points, with the system exhibiting bistability in the region located between these lines. The system loses bistability when the two curves converge at the cusp point, marking the transition to a single stable state. The horizontal line corresponds to the RFP resource competitiveness value used in panels B and C.

A bimodal distribution often signals bistability, typically arising from an underlying positive feedback loop within the system [23]. To identify the implicit positive feedback, we mapped out the hidden interactions introduced by resource competition, depicted as the orange lines in **Fig 3A**. That is, while Fig 3A represents the same system as Fig 2A, it highlights the indirect regulatory effects arising from resource competition. Beyond the direct inhibition in the gene cascade—where GFP protein expression inhibits the production of RFP mRNA—additional inhibitory interactions now occur on the transcription and translation processes of both genes, affecting each gene’s own production as well as that of its counterpart. Notably, inhibition from RFP mRNA on GFP protein translation creates a double-negative feedback loop with the direct inhibition GFP protein exerts on RFP mRNA, effectively forming a positive feedback mechanism in the system.

To determine whether this implicit positive feedback can indeed generate bistability, we conducted a one-parameter bifurcation analysis to examine how the steady-state values of GFP and RFP change with parameter $I_{1}$ (**Fig 3B**). The analysis revealed that the system exhibits bistability within the range $0.87<I_{1}<1.1$, aligning with the thresholds observed in previous stochastic analyses. Likewise, bistability was found when varying the dissociation constant of GFP-mediated inhibition on RFP translation, with a bistable range of $15.2<K_{g}<18.9$ (S4 Fig). To assess the sensitivity of our results to parameter variations, we conducted a new parameter sensitivity analysis. Specifically, we performed one-parameter bifurcation analyses, considering a 20% increase or decrease in each parameter. Consistently, bistability was observed across these parameter variations (S5 Fig). We quantified the relative changes in the saddle-node bifurcation points due to parameter variations and found that the system is particularly sensitive to parameters involved in the proposed hidden positive feedback loop (Fig 3C and 3D). For example, parameters that influence either the total level of GFP or the inhibition strength of RFP mRNA by GFP (such as k_p1_, d_p1_, n, k_m1_, d_m1_, and K_g_) are critical for the first branch of the positive feedback loop. Parameters that affect either mRNA levels or resource competition for translation (such as k_m2_, d_m2_, J_p2_, and I_2_) are essential for the second branch of the positive feedback loop. In contrast, parameters outside this loop, such as k_p2_, d_p2_ (which determine RFP production) and J_m1_ and J_m2_ (associated with resource competition for transcription), do not alter the bistability range. These findings not only highlight the pivotal role of the hidden feedback loop components in shaping the system’s bistability but also offer valuable insights for designing experiments to validate these predictions. Specifically, tuning the parameters mentioned above could modulate the system’s bistable behavior, providing a targeted approach for experimental verification and control.

Nullcline analysis reveals three steady states at the nullcline intersection points (**Fig 3E**). One is stable, with GFP expression low and RFP expression high (**Fig 3E**, left solid circle), another is stable with RFP expression low and GFP expression high (**Fig 3E**, right solid circle), and the third is unstable, situated between the two stable steady states (**Fig 3E**, open circle). We further performed two-parameter bifurcation analysis to investigate how GFP inducer dose and RFP resource competition (1/J_P2_) influence the stable states. As shown in **Fig 3F**, increasing RFP resource competition expands the range of bistability. However, if RFP resource competition falls below a critical threshold (the cusp point), the bistable system ceases to exist. At this point, the implicit inhibition of GFP translation by rfp mRNA weakens, and the positive feedback loop mediated by resource competition is no longer strong enough to sustain bistability. The sensitivity of the bistable region in the two-parameter bifurcation diagram to parameter variations (S6 Fig) aligns with the results observed in the one-parameter bifurcation analysis. Taken together, the stochastic switching between GFP-high/RFP-low and GFP-low/RFP-high states is driven by resource competition.

### Removal of resource competitive noise deescalates noise level

Our previous work demonstrated that resource competition introduces additional noise to a gene, which arises from fluctuations in the opposing mRNA. This type of noise is termed “resource competitive noise” [20]. To isolate the effect of resource competition without the contribution from resource competitive noise, we set the opposing mRNA to its mean value rather than using its real-time stochastic value [20]. This modification preserves the same level of resource competition but removes the noise due to the opposing mRNA fluctuations.

**Fig 4:**
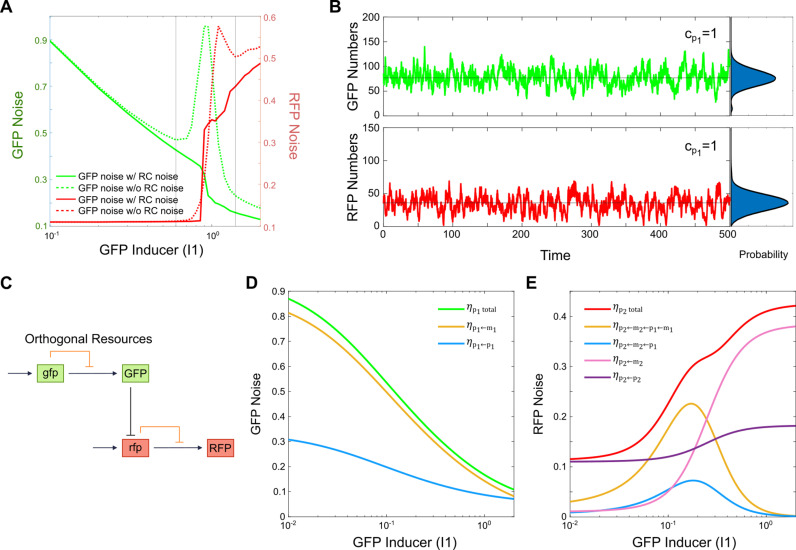
Controlling state switching by mitigating resource competition. A) Total protein noise as a function of GFP inducer dose ($I_{1}$) when resource competitive noise is removed. B) Stochastic trajectory of GFP and RFP protein levels for an GFP inducer dose ($I_{1}$) within the bistable range, with resource competitive noise removed. Horizontal lines represent local modes in the protein distributions, indicating the values at which the system occurs most frequently, and probability distributions of protein levels are shown on the right. C) Schematic of a genetic inhibition cascade circuit with orthogonal resources. D) GFP total noise and its components as a function of GFP inducer dose ($I_{1}$) in the orthogonal resource scenario. E) RFP total noise and its components as a function of in GFP inducer dose ($I_{1}$) under orthogonal resources.

As shown in **Fig 4A**, compared to the system with resource competition (dotted lines), removing the competitive noise causes the characteristic humps in protein noise to disappear, indicating the loss of the increased noise around the threshold. Additionally, we observed noise amplification in the monostable range near the bistable region, which is attributed to transient state transitions arising from inherent stochasticity. This behavior is apparent in the stochastic trajectories. At a GFP inducer dose within the bistable region ($I_{1}=1$), fluctuations in both GFP and RFP no longer lead to state switching between the bistable states (**Fig 4B**). Instead, GFP expression fluctuates only around the high GFP mode, while RFP expression fluctuates only around the low RFP mode. Therefore, resource competitive noise is responsible for stochastic state switching and the enhancement of noise in the system. By removing this noise, the state switching behavior is suppressed, and the previously observed noise peaks in both GFP and RFP are eliminated.

Alternative strategy to mitigate resource competition effects *in vivo* is to utilize orthogonal resources [24–27]. By creating separate pools of transcriptional and translational resources, this approach removes the double-negative feedback loop, as illustrated in **Fig 4C**. Orthogonal resources have been shown to reduce noise in gene circuits by limiting the resources allocated to transcription and translation for each gene independently, avoiding competition [20]. For the cascading two-gene system with orthogonal resources, the analytical solution resembles that of the system with unlimited resources (see Method Section for details). As shown in **Fig 4D**, the GFP total noise and its components follow a pattern similar to the previous analysis with unlimited resources. However, in the RFP noise analysis, orthogonal resource constraints significantly reduce noise propagation from GFP mRNA to RFP (**Fig 4E**), eliminating the large noise peak seen in the unlimited resources scenario (**Fig 1D**). To directly compare the noise behavior between the orthogonal and shared resource systems, the GFP and RFP total noise is overlayed in S7 Fig GFP total noise is significantly lower in the orthogonal resource system compared to the shared resource system, particularly in the regions where noise humps were observed. This overall noise reduction is due to the enhanced expression of GFP in the orthogonal system, while the disappearance of the noise humps is attributed to the removal of resource competition noise and its associated stochastic switching. Interestingly, the RFP noise is much higher in the orthogonal resource system compared to the shared resource system before the appearance of noise humps in the latter. This is due to the reduced expression level of RFP as more GFP is produced instead. However, we still observe the disappearance of the noise humps and reduction in RFP noise, which is consistent with our conclusion that resource competition induces state stochastic switching and noise amplification. It is noted that the efficiency of orthogonal systems is a critical factor that can influence system noise. Studies have demonstrated that an insufficient number of orthogonal ribosomes leads to reduced protein production, which is associated with increased noise [27]. In other words, while orthogonal systems can effectively decouple gene expression and mitigate resource competition noise, ensuring an adequate supply of orthogonal resources is crucial for minimizing competition-independent noise. In summary, these findings confirm that resource competition can indeed drive state switching and amplify gene expression noise.

## Discussion

This work highlights the critical role of resource competition in shaping gene expression noise. We show that incorporating resource competition into a simple regulatory cascade effectively introduces a double-negative feedback loop, revealing hidden feedback mechanisms that significantly alter both deterministic and stochastic system behaviors. Remarkably, this implicit feedback can result in bistability, where stable states emerge unexpectedly due to resource-mediated interactions. Moreover, resource competition generates additional noise that facilitates stochastic switching between these stable states, allowing only one gene to be highly expressed at a time. Consequently, noise propagation dynamics in simple cascade circuits are dramatically altered, highlighting the importance of accounting for resource competition in synthetic gene circuit design. The interplay between resource competition and noise not only complicates the engineering of robust gene circuits but can also lead to highly nonintuitive system behaviors, emphasizing the challenges and opportunities in designing predictable and resilient synthetic systems.

To experimentally validate our predictions, a library of cascade gene circuits can be constructed and tested in *E. coli* where ribosomal resources are limited for synthetic gene circuits. Fine-tuning promoter and RBS strength combinations will be essential to amplifying the hidden positive feedback sufficiently to achieve bistability. These efforts will pave the way for leveraging resource competition in the design of synthetic gene circuits, such as memory elements and toggle switches. Furthermore, the observed noise behavior holds promise for applications in developing biosensors or biomarkers.

Previously, WTA behavior was observed in circuits with two self-activation modules, highlighting the role of positive feedback in driving this type of resource competition [16]. In this work, however, we uncover an alternative mechanism for WTA behavior that arises from resource competition. This mechanism involves no inherent feedback loop in the circuit design; instead, an implicit feedback loop emerges from the combination of a negative regulation mediated by resource competition and another built into the circuit itself. This setup may represent a simpler mechanism for WTA driven by resource competition. Testing this experimentally and investigating other possible mechanisms of nonlinear resource competition could be fascinating directions for future research.

Various control strategies have been proposed to address the impact of resource competition on the deterministic behavior of synthetic gene circuits, including negative feedback loops and feedforward loops [26–36]. Here, we demonstrated that eliminating resource competitive noise can reduce stochastic switching, thereby controlling overall noise levels. Additionally, using orthogonal resources prevents the emergence of both bistability and associated stochastic switching. In our prior work, we tested different types of controllers—global, local, NCR negative feedback, and antithetic controllers—to regulate gene expression noise in a simple two-gene system under resource competition [20,37] to regulate the gene expression noise levels in simple two-gene system under the context of resource competition. Exploring other feedback and feedforward control strategies in the context of noise propagation would be compelling. For instance, introducing additional negative feedback is expected to reduce the bistable range [38], thereby minimizing stochastic switching and lowering noise levels.

## Methods

### Mathematical model for two-gene cascade circuit under different resource conditions

A two-gene circuit was used to show the propagation of noise through inhibition. The two genes are differentiated between the fluorescent protein labels GFP and RFP, where the first gene (GFP) directly inhibits the second (RFP). Three separate scenarios are considered for this gene circuit: unlimited resources as shown in Fig 1A, resource competition as shown in Fig 2A or Fig 3A, and orthogonal resources as shown in Fig 4C. As in Goetz et al. [20], the system is generally described by a set of ordinary differential equations:


$$\frac{dm_{i}}{dt}=J_{m_{i}}^{+}-J_{m_{i}}^{-}$$

$$\frac{dp_{i}}{dt}=J_{p_{i}}^{+}-J_{p_{i}}^{-}$$

where $m_{i}$ and $p_{i}$ represent the concentration of the mRNA and protein in module *i*.

#### Model for the system with unlimited resources.

A system with unlimited resources has absolutely no competition and can be modeled by.


$$\frac{dm_{i}}{dt}=k_{m_{0i}}+k_{m_{i}}R_{i}-d_{m_{i}}m_{i},$$

$$\frac{dp_{i}}{dt}=k_{p_{i}}m_{i}-d_{p_{i}}p_{i}$$

with


$$R_{1}=I_{1},$$

$$R_{2}=\frac{I_{2}K_{g}^{n}}{\left(p_{1}^{n}+K_{g}^{n}\right)}$$

where $k_{m_{0i}}$ with the subscripts i=1,2 are the basal transcription rate of GFP and RFP, respectively; $k_{m_{i}}$ and $k_{p_{i}}$ are the transcription and translation rate constants, and $d_{m_{i}}$ and $d_{p_{i}}$ are the degradation rate constants for the mRNAs and proteins, respectively. Parameters $I_{i}$ represent the inducer doses for two genes. The Inhibitive Hill function is used in $R_{2}$ to represent the inhibition of rfp transcription by GFP. $K_{g}$ is the dissociation constant, and n is the Hill coefficient representing the nonlinearity of the inhibition. Thus, $J_{m_{i}}^{+}=k_{m_{0i}}+k_{m_{i}}R_{1}$ and $J_{p_{i}}^{+}=k_{p_{i}}m_{i}$ while $J_{m_{i}}^{-}=d_{m_{i}}m_{i}$ and $J_{p_{i}}^{-}=d_{p_{i}}p_{i}$. Setting these derivatives to zero gives the steady-state mRNA and protein mean concentrations, respectively, as


$$\left\langle m_{i}\right\rangle =\frac{{k_{m_{0i}}+k}_{m_{i}}R_{i}}{d_{m_{i}}},$$

$$\left\langle p_{i}\right\rangle =\frac{k_{p_{i}}\left\langle m_{i}\right\rangle }{d_{p_{i}}}.$$

The deterministic simulation shown in Figs 1, S1 and S2 is based on this model.

#### Model for the system with limited shared resources.

In a system with limited shared resources, resource competition arises as genes compete both with themselves and with each other for the constrained transcriptional and translational machinery. The equations for this system are


$$\frac{dm_{i}}{dt}=\frac{k_{m_{0i}}+k_{m_{i}}R_{i}}{1+\sum_{j}\frac{R_{j}}{J_{m_{j}}}}-d_{m_{i}}m_{i},$$

$$\frac{dp_{i}}{dt}=\frac{k_{p_{i}}m_{i}}{1+\sum_{j}\frac{m_{j}}{J_{p_{j}}}}-d_{p_{i}}p_{i}$$

where $J_{m_{i}}$ and $J_{p_{i}}$ are the effective transcriptional and translational capacities of limited resources in the host cell for synthetic gene circuits, respectively. The resource competition model is similarly defined in Zhang et al. [16], as well as Goetz et al. [20] Note that as $J_{m_{i}}$ and $J_{p_{i}}$ approaches infinity, the resource competition model reduces the unlimited resources model, so


$$J_{m_{i}}^{+}=\frac{k_{m_{0i}}+k_{m_{i}}R_{i}}{1+\sum_{j}\frac{R_{j}}{J_{m_{j}}}},$$

$$J_{p_{i}}^{+}=\frac{k_{p_{i}}m_{i}}{1+\sum_{j}\frac{m_{j}}{J_{p_{j}}}}.$$

Again, setting these derivatives to zero yields the steady state mRNA and protein mean concentrations as


$$\left\langle m_{i}\right\rangle =\frac{k_{m_{0i}}+k_{m_{i}}R_{i}}{d_{m_{i}}\left(1+\sum_{j}\frac{R_{j}}{J_{m_{j}}}\right)},$$

$$\left\langle p_{i}\right\rangle =\frac{k_{p_{i}}\left\langle m_{i}\right\rangle }{d_{p_{i}}\left(1+\sum_{j}\frac{\left\langle m_{j}\right\rangle }{J_{p_{j}}}\right)}.$$

The deterministic simulation shown in Figs 3 and S3–S6 is based on this model.

#### Model for the system with limited orthogonal resources.

A system with orthogonal resources eliminates the resource competition between genes, where the two genes still compete with themselves due to the limitation in the resources but not with each other. The system is described by.


$$\frac{dm_{i}}{dt}=\frac{k_{m_{0i}}+k_{m_{i}}R_{i}}{1+\frac{R_{i}}{J_{m_{i}}}}-d_{m_{i}}m_{i},$$

$$\frac{dp_{i}}{dt}=\frac{k_{p_{i}}m_{i}}{1+\frac{m_{i}}{J_{p_{i}}}}-d_{p_{i}}p_{i}$$

with $J_{m_{i}}^{+}=\frac{k_{m_{0i}}+k_{m_{i}}R_{i}}{1+\frac{R_{i}}{J_{m_{i}}}}$ and $J_{p_{i}}^{+}=\frac{k_{p_{i}}m_{i}}{1+\frac{m_{i}}{J_{p_{i}}}}$. In the steady state, the mRNA and protein mean concentrations are, respectively,


$$\left\langle m_{i}\right\rangle =\frac{k_{m_{0i}}+k_{m_{i}}R_{i}}{d_{m_{i}}\left(1+\frac{R_{i}}{J_{m_{i}}}\right)},$$

$$\left\langle p_{i}\right\rangle =\frac{k_{p_{i}}\left\langle m_{i}\right\rangle }{d_{p_{i}}\left(1+\frac{\left\langle m_{i}\right\rangle }{J_{p_{i}}}\right)}.$$

The deterministic simulation shown in Figs 4 and S7 is based on this model.

### Gillespie stochastic simulations

To stimulate the stochastic trajectories of mRNA and protein levels, the standard Gillespie algorithm is used [39]. The detailed steps can be found in our previous work [20]. The simulation shown in Figs 2,4, and S3 is based on this method.

#### Analytical solution for noise level from the normalized fluctuation-dissipation theorem.

Noise (η) is defined for both mRNAs and proteins as the coefficient of variation:


$$\eta_{i}=\sqrt{\frac{\sigma_{ii}}{\mu_{i}^{2}}}=\frac{\sigma_{i}}{\mu_{i}}$$

and was solved for using the Fluctuation-Dissipation Theorem as described in Goetz et al. [20]

#### Analytical solution for the case without resource competition.

For the unlimited resources and orthogonal resources systems, the matrix ℳ and 𝒟 are given by


$$M=[\begin{array}{cccc} \frac{H_{11}}{\tau_{1}} & 0 & 0 & 0\\ \frac{H_{21}}{\tau_{2}} & \frac{H_{22}}{\tau_{2}} & 0 & 0\\ 0 & \frac{H_{32}}{\tau_{3}} & \frac{H_{33}}{\tau_{3}} & 0\\ 0 & 0 & \frac{H_{43}}{\tau_{4}} & \frac{H_{44}}{\tau_{4}} \end{array}],\;D=[\begin{array}{cccc} \frac{2}{m_{1}\tau_{1}} & 0 & 0 & 0\\ 0 & \frac{2}{p_{1}\tau_{2}} & 0 & 0\\ 0 & 0 & \frac{2}{m_{2}\tau_{3}} & 0\\ 0 & 0 & 0 & \frac{2}{p_{2}\tau_{4}} \end{array}].$$

The normalized FDT noise equations are then solved with the following H equations.


$$H_{11}=\frac{\partial \ln\left(\frac{J_{m_{1}}^{-}}{J_{m_{1}}^{+}}\right)}{\partial \ln\left(M_{1}\right)},\;H_{22}=\frac{\partial \ln\left(\frac{J_{p_{1}}^{-}}{J_{p_{1}}^{+}}\right)}{\partial \ln\left(P_{1}\right)},\;H_{33}=\frac{\partial \ln\left(\frac{J_{m_{2}}^{-}}{J_{m_{2}}^{+}}\right)}{\partial \ln\left(M_{2}\right)},\;H_{44}=\frac{\partial \ln\left(\frac{J_{p_{2}}^{-}}{J_{p_{2}}^{+}}\right)}{\partial \ln\left(P_{2}\right)},$$

$$H_{21}=\frac{\partial \ln\left(\frac{J_{p_{1}}^{-}}{J_{p_{1}}^{+}}\right)}{\partial \ln\left(M_{1}\right)},\;H_{32}=\frac{\partial \ln\left(\frac{J_{m_{2}}^{-}}{J_{m_{2}}^{+}}\right)}{\partial \ln\left(P_{1}\right)},\;H_{43}=\frac{\partial \ln\left(\frac{J_{p_{2}}^{-}}{J_{p_{2}}^{+}}\right)}{\partial \ln\left(M_{2}\right)}\;.$$

For the unlimited resource system, there is.


$$J_{m_{i}}^{+}=k_{m_{0i}}+k_{m_{i}}R_{i}\Omega ,\;J_{m_{i}}^{-}=M_{i}d_{m_{i}},$$

$$J_{p_{i}}^{+}=k_{p_{i}}M_{i}\Omega ,\;J_{p_{i}}^{-}=P_{i}d_{p_{i}}.$$

The noise calculation in S1 and S2 Figs are based on this method.

#### Analytical solution for the case with orthogonal competition.

For the orthogonal resource system, there is


$$J_{m_{i}}^{+}=\frac{k_{m_{0i}}+k_{m_{i}}R_{i}}{1+\frac{R_{i}}{J_{m_{i}}}\;}\Omega ,\;J_{m_{i}}^{-}=M_{i}d_{m_{i}},$$

$$J_{p_{i}}^{+}=\frac{k_{p_{i}}M_{i}}{1+\frac{{(M}_{i}/\Omega )}{J_{p_{i}}}}\Omega ,\;J_{p_{i}}^{-}=P_{i}d_{p_{i}}.$$

Substituting $H_{11}=1,\;H_{22}=1,\;H_{33}=1$, and $H_{44}=1$, the noise equations become


$$\eta_{m_{1}\;total}^{2}=\overset{\eta_{m_{1}\leftarrow m_{1}}^{2}}{\overbrace{\frac{1}{M_{1}}}},$$

$$\eta_{p_{1}\;total}^{2}=\overset{\eta_{p_{1}\leftarrow p_{1}}^{2}}{\overbrace{\frac{1}{P_{1}}}}+\overset{\eta_{p_{1}\leftarrow m_{1}}^{2}}{\overbrace{\eta_{m_{1}\leftarrow m_{1}}^{2}H_{21}^{2}\frac{\frac{1}{\tau_{2}}}{\frac{1}{\tau_{1}}+\frac{1}{\tau_{2}}}}},$$

$$\eta_{m_{2}\;total}^{2}=\overset{\eta_{m_{2}\leftarrow m_{2}}^{2}}{\overbrace{\frac{1}{M_{2}}}}+\overset{\eta_{m_{2}\leftarrow p_{1}}^{2}}{\overbrace{\eta_{p_{1}\leftarrow p_{1}}^{2}H_{32}^{2}\frac{\frac{1}{\tau_{3}}}{\frac{1}{\tau_{2}}+\frac{1}{\tau_{3}}}}}+\overset{\eta_{m_{2}\leftarrow p_{1}\leftarrow m_{1}}^{2}}{\overbrace{\eta_{p_{1}\leftarrow m_{1}}^{2}\left(1+\frac{\frac{1}{\tau_{2}}}{\frac{1}{\tau_{1}}+\frac{1}{\tau_{3}}}\right)H_{32}^{2}\frac{\frac{1}{\tau_{3}}}{\frac{1}{\tau_{2}}+\frac{1}{\tau_{3}}}}},$$

$$\eta_{p_{2}\;total}^{2}=\overset{\eta_{p_{2}\leftarrow p_{2}}^{2}}{\overbrace{\frac{1}{P_{2}}}}+\overset{\eta_{p_{2}\leftarrow m_{2}}^{2}}{\overbrace{\eta_{m_{2}\leftarrow m_{2}}^{2}H_{43}^{2}\frac{\frac{1}{\tau_{4}}}{\frac{1}{\tau_{3}}+\frac{1}{\tau_{4}}}}}+\overset{\eta_{p_{2}\leftarrow m_{2}\leftarrow p_{1}}^{2}}{\overbrace{\eta_{m_{2}\leftarrow p_{1}}^{2}\left(1+\frac{\frac{1}{\tau_{3}}}{\frac{1}{\tau_{2}}+\frac{1}{\tau_{4}}}\right)H_{43}^{2}\frac{\frac{1}{\tau_{4}}}{\frac{1}{\tau_{3}}+\frac{1}{\tau_{4}}}}}+$$

$$\overset{\eta_{p_{2}\leftarrow m_{2}\leftarrow p_{1}\leftarrow m_{1}}^{2}}{\overbrace{\eta_{m_{2}\leftarrow p_{1}\leftarrow m_{1}}^{2}\left(1+\frac{\frac{1}{\tau_{3}}}{\frac{1}{\tau_{2}}+\frac{1}{\tau_{4}}}\left(1+\frac{\frac{1}{\tau_{2}}}{\frac{1}{\tau_{1}}+\frac{1}{\tau_{2}}+\frac{1}{\tau_{3}}}\cdot \frac{\frac{1}{\tau_{2}}+\frac{1}{\tau_{3}}}{\frac{1}{\tau_{1}}+\frac{1}{\tau_{4}}}\right)\right)H_{43}^{2}\frac{\frac{1}{\tau_{4}}}{\frac{1}{\tau_{3}}+\frac{1}{\tau_{4}}}}},$$

where $\eta_{\alpha \leftarrow \beta }^{2}$ represents noise propagated from β to α.

The simulation in Figs 4 and S7 are based on this method.

### Model parameters

Unless otherwise varied, parameters used are $k_{m_{01}}=0.4$, $k_{m_{1}}=8$, $k_{p_{1}}=15$, $d_{m_{1}}=1$, $d_{p_{1}}=1$, $I_{1}=1$, $J_{m_{1}}=40$, $J_{p_{2}}=20$, $k_{m_{02}}=1$, $k_{m_{2}}=25$, $k_{p_{2}}=30$, $d_{m_{2}}=1$, $d_{p_{2}}=1$, $I_{2}=1$, $J_{m_{2}}=40$, $J_{p_{2}}=2$, n=3, $K_{g}=17$, and Ω=1.5. For the one-parameter and two-parameter bifurcation analysis, the GFP inducer dose ($I_{1}$) and dissociation constant of GFP-mediated inhibition on RFP translation ($K_{g}$) varied in range, i.e., $0\leq I_{1}\leq 2$, $1\leq K_{g}\leq 40$. In addition, RFP resource competitivity ($1/J_{p_{2}}$) was varied in a range $0\leq 1/J_{p_{2}}\leq 1$ in the two-parameter bifurcation analysis. The parameters are primarily based on the assumption of significant resource competition within the system, designed to mimic experimental findings. The production and degradation rates are set relative to one another to ensure that mRNA and protein levels fall within a biologically relevant range, while also exhibiting significant noise. The resource competition parameters are then adjusted according to the mean mRNA number to ensure the competition is as pronounced as observed in experiments [13,15,16,27]. Experimentally, these parameters can be tuned by varying the strength of promoters and ribosome binding sites (RBS). It's important to note that the conclusions are not highly sensitive to specific parameter choices, as demonstrated by the sensitivity analysis. Additionally, the dependence on key parameters was analyzed using bifurcation analysis, along with a local parameter sensitivity analysis, providing valuable insights for future experimental design.

## Supporting information

S1 FigThe number of gfp and rfp mRNAs with increase of GFP inducer dose ($I_{1}$).(TIF)

S2 FigRFP noise levels with changes of dissociation constant of GFP-mediated inhibition on RFP translation ($K_{g}$).RFP total noise level and its four components on GFP inducer dose ($I_{1}$) with (A) $K_{g}=10$ and (B) $K_{g}=30$.(TIF)

S3 FigInduced stochastic state switching by resource competition perturbs the noise propagation.A) The mean numbers of and B) total noise of GFP and RFP as the dissociation constant of GFP-mediated inhibition on RFP translation ($K_{g}$) increases. C) Stochastic trajectories of GFP and RFP protein numbers for $K_{g}$ values. Horizontal lines represent local protein mode(s) in the system. Probability of protein numbers along the right side of the trajectories.(TIF)

S4 FigThe one-parameter bifurcation diagram of GFP and RFP in terms of dissociation constant of GFP-mediated inhibition on RFP translation ($K_{g}$).(TIF)

S5 FigSensitivity analysis of the one-parameter bifurcation.Each panel shows how the steady-state level of GFP protein with changes in the inducer dose ($I_{1}$) varies when each model parameter is either increased (red) or decreased (blue) by 20% relative to the baseline parameter set. The blue curve represents the bifurcation diagram for the baseline parameter set, providing a comparison for the altered conditions.(TIF)

S6 Fig Sensitivity analysis of the two-parameter bifurcation. Each panel shows how the saddle-node bifurcation points varies when each model parameter is either increased (red) or decreased (blue) by 20% relative to the baseline parameter set. The blue curve represents the two-parameter bifurcation diagram for the baseline parameter set, providing a comparison for the altered conditions.
(TIF)

S7 FigComparison of total protein noise between the orthogonal and shared resource systems.A) Total protein noise as a function of GFP inducer dose ($I_{1}$) with (dashed line) or without (solid line) resource competition noise. B) Total protein noise as a function of RFP inducer dose ($I_{1}$) with (dashed line) or without (solid line) resource competition noise.(TIF)
